# Proton pump inhibitors and the risk of pneumonia: a comparison of cohort and self-controlled case series designs

**DOI:** 10.1186/1471-2288-13-82

**Published:** 2013-06-24

**Authors:** Emmae N Ramsay, Nicole L Pratt, Philip Ryan, Elizabeth E Roughead

**Affiliations:** 1 Data Management and Analysis Centre, Discipline of Public Health, University of Adelaide, Adelaide, Australia; 2 School of Pharmacy and Medical Sciences, Quality Use of Medicines and Pharmacy Research Centre; Sansom Institute, University of South Australia, Adelaide, Australia

## Abstract

**Background:**

To compare the results of a new-user cohort study design and the self-controlled case series (SCCS) design using the risk of hospitalisation for pneumonia in those dispensed proton pump inhibitors compared to those unexposed as a case study.

**Methods:**

The Australian Government Department of Veterans’ Affairs administrative claims database was used. Exposure to proton pump inhibitors and hospitalisations for pneumonia were identified over a 4 year study period 01 Jul 2007 -30 Jun 2011. The same inclusion and exclusion criteria were applied to both studies, however, the SCCS study included subjects with a least one hospitalisation for pneumonia.

**Results:**

There were 105,467 subjects included in the cohort study and 6775 in the SCCS. Both studies showed an increased risk of hospitalisations for pneumonia in the three defined risk periods following initiation of proton pump inhibitors compared to baseline. With the highest risk in the first 1 to 7 days (Cohort RR, 3.24; 95% CI (2.50, 4.19): SCCS: RR, 3.07; 95% CI (2.69, 3.50)).

**Conclusions:**

This study has shown that the self-controlled case series method produces similar risk estimates to a new-users cohort study design when applied to the association of proton pump inhibitors and pneumonia. Exposure to a proton pump inhibitor increases the likelihood of being admitted to hospital for pneumonia, with the risk highest in the first week of treatment.

## Background

Observational studies provide important information about the safety and effectiveness of medicines. However, these designs are often criticised due to a lack of control for unmeasured confounding. These problems are amplified when administrative databases are used, since data were not collected for purposes of research and potentially important clinical and patient demographic data are often absent.

Case-only designs [1] have been suggested as an alternative to more traditional observational studies such as the case–control and the cohort study as they have the potential to control for fixed patient specific confounders even those that are unmeasured. One such design is the self-controlled case series [2,3], which minimises confounding by means of its within-subject design, meaning that the patient is used as their own control [2,3]. A cohort study compares patients who were exposed to patients who were not exposed, whereby all confounding needs to be controlled for numerically, however the self-controlled case series compares the number of events in periods of exposure with the number of events in periods of non-exposure in the same person. The self-controlled case series design controls implicitly for fixed known and unknown confounders that do not vary over time, such as genetic and socio-economic factors, while time varying confounders can be adjusted within the model [2,3]. An advantage of the self-controlled case series design is that it requires only those individuals who have had the event of interest which results in reduced computational time.

The self-controlled case series method was developed to study adverse events associated with vaccines where use of the vaccine is transient and the adverse event is acute. It has been compared previously to the cohort study design to evaluate vaccine safety [4]. This method originates from cohort logic and the emphasis like a cohort study is on the relative incidence or relative hazard of an event [2]. The method has not been widely applied in pharmacoepidemiological research to study acute effects of transient medicine exposures and there has been only limited research to compare the findings of the self-controlled case series with cohort study designs in the field of pharmacoepidemiology [5].

In this study we aimed to compare the two study designs using the example of the association between proton pump inhibitors and community acquired pneumonia. This example was chosen because the outcome, pneumonia, is acute [6-8] and is at its highest risk within 7 days of initiation of a proton-pump inhibitor [6]. Further, exposure to proton pump inhibitors is often transient, but may be chronic. Previous work conducted in the database used for the present study found that 32% of new users of proton pump inhibitors had discontinued by 8 weeks, while 62% had discontinued within 12 months [9]. Treatment duration was found to be longer for those initiated in hospital (195 days) than those initiated by a GP (124 days) [9]. The study identified that there was a mix of long and short term use suggestive of the treatment nature of proton pump inhibitors. The objective of this study was to compare the results of a new-user cohort study design and the self-controlled case series design using the risk of hospitalization for pneumonia in those dispensed proton pump inhibitors as a case-study.

## Methods

The data source for this study was the Australian Department of Veterans’ Affairs (DVA) administrative claims databases. DVA claims data contain records of prescription medicines dispensed under the Pharmaceutical Benefits Scheme and Repatriation Pharmaceutical Benefits Scheme, medical and allied health services and hospital admissions provided to subjects for whom DVA pays a subsidy. The treatment population is approximately 310,000 subjects, and there are approximately 100 million pharmacy records, 200 million medical and allied health service records and over 6 million hospital admission records. A client file is maintained by DVA which includes data on gender, date of birth, date of death and family status. We undertook two study designs using the same populations and study inclusion and exclusion criteria. The study period was the 1st July 2007 to the 30th June 2011. In both studies eligible subjects were those 65 years of age or over at the 1st July 2007, who had a least one medication prescribed in the 6 months prior to entry into the cohort and who were eligible for all health services subsidised by DVA. Entry into both studies was the 1^st^ July 2007. Subjects were excluded if they had been prescribed a proton pump inhibitor in the 12 months prior to the study start or a histamine 2 receptor antagonist (H2RA) in the six months prior to or during the study. The reason for excluding H2RA is because we were interested in the more potent medicine, proton pump inhibitors and including H2RAs could have diluted the effect if it exists with proton pump inhibitors.

For both studies, exposure was determined by identifying prescription data on proton pump inhibitors (omeprazole, pantoprazole, lansoprazole, rabeprazole, esomeprazole) and the outcome of interest was a primary diagnosis of pneumonia (ICD-10: J12, J13, J14, J15, J16, J17, J18) during the study period.

Dosage information was not available in the data set so duration of proton pump inhibitor use was defined as the period within which 75% of subjects returned for a repeat dispensing of the medicine. This was calculated using the entire database as 36 days. This is consistent with the standard package size of proton pump inhibitors of 28–30 tablets, taken once daily. Exposure to proton pump inhibitors was defined as the date from when a prescription was dispensed plus 72 days (1 duration interval of 36 days plus a grace period of 36 days). Any person-time in the study prior to a subject’s first prescription was considered unexposed time. Subjects with no record of dispensing of a proton pump inhibitor for more than 72 days after their last dispensing were considered unexposed to a proton pump inhibitor from 72 days after their last dispensing. This was to allow for possible non-compliance and stock piling of medicine. Therefore, for both studies exposure to a proton pump inhibitor is a time dependent variable. Inclusion of unexposed time prior to proton pump inhibitors initiation helps to prevent immortal time bias being a factor in this study design [10].

For both studies, the time after initiation of a proton pump inhibitor was stratified into *a priori* risk periods: 1 to 7 days, 8 to 30 days and greater than 30 days. The actual day of initiation was excluded from the analysis because in cases where the pneumonia hospitalisation occurred on the same day it was not possible to determine which occurred first.

For the cohort study, eligible subjects were followed until death, pneumonia hospitalization or study end (30 June 2011). The numbers of hospitalisations for pneumonia during exposed and non-exposed times were determined. Individuals could be unexposed for the whole study or they could have unexposed periods before and after exposure to a proton pump inhibitor. Hospitalisation rates were calculated as the cumulative number of hospitalisations in each period divided by the number of days at risk. Rate Ratios were calculated using Poisson generalised estimating equations (GEE) to allow for clustering of observations within patients, adjusting for both fixed and time-varying confounders. Fixed confounders were assessed at study entry; including age, gender, socioeconomic index of disadvantage for area of residence [11]. The following time-varying confounders were determined annually; number of co-morbidities (using the validated Rx-Risk-V [12] score), number of prescriptions, number of prescribers, number of pharmacies and number of occupational therapy visits and speech pathology services. The remaining time-varying confounders changed as the season changed (season), when subjects entered aged care (residential aged-care status) and when they had their first script of tiotropium as a proxy indicator of chronic obstructive pulmonary disease (COPD), and first concurrent use of angiotensin renin system medicines with frusemide as a proxy indicator of those with heart failure. All the above confounders were included in the model and the decision to include them was based on clinical knowledge.

**Figure 1:**
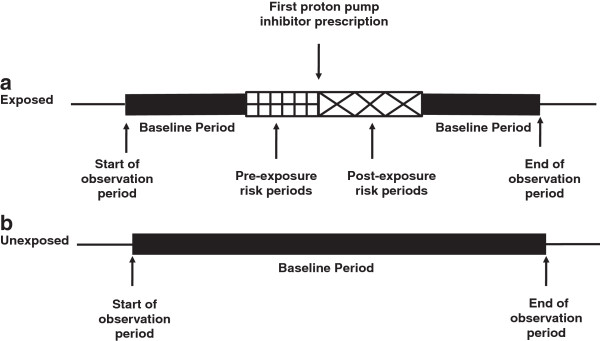
**A graphical representation of the self**-**controlled case**-**series design for patients (a) exposed to a proton pump inhibitor (b) unexposed to a proton pump inhibitor.**

For the self-controlled case series study, eligible subjects were all subjects who had a hospitalisation with a primary diagnosis of pneumonia during the study period between 1st January 2007 and 30th June 2011. These are the same subjects who were identified in the cohort study as having a hospitalisation for pneumonia. Exposure to proton pump inhibitors was calculated and time partitioned into unexposed time and pre and post exposure times. Subjects with a pneumonia hospitalisation but with no record of being prescribed a proton pump inhibitor were included to adjust for the change in the underlying hospitalisation rate associated with age [2]. In addition to the post-exposure risk periods, two consecutive 30 day (1–30, 31–60 days) and a 60 day (61–120 days) pre-exposure risk periods were included prior to proton pump inhibitor initiation; to allow for time periods where proton pump inhibitors may have been initiated as a result of hospitalisation. The consequence of not partitioning this prior exposure would be an increase in the rate of pneumonia hospitalisations in the unexposed period. This would cause a bias towards the null in the exposed resulting in a decrease in the rate ratios in the post exposure periods. All residual time before and after exposure was considered unexposed and used for the baseline comparison. In each risk period, the cumulative number of hospitalisations was divided by the person-years at risk and these were compared to the risk in the baseline period (Figure 1). If a subject was re-hospitalised within 30 days, the subsequent hospitalisation(s) were excluded as they were considered to be related and part of the same episode [2]. Rate ratios were calculated using conditional Poisson regression, with results presented as adjusted rate ratios and 95% confidence intervals. We also performed two sensitivity analyses for the self-controlled case series 1) adjusted for the same time-varying confounders as the cohort study, 2) analysis restricted to patients alive at hospital discharge. The self-controlled case series design controls implicitly for fixed covariates [2,3], however, all SCCS analyses were adjusted for time-varying age and study year. All analyses were performed using SAS version 9.12 (SAS Institute, Cary, NC). This research has ethics approval from the Department of Veteran Affairs human research committee and the University of South Australia human research committee.

## Results

### Cohort study

The final cohort consisted of 105,467 subjects, of whom 51.5% were male. Their median age was 83 years (range 80–86) and 11.2% entered a residential aged-care facility during the study (Table 1).

**Table 1:** Demographics of the Cohort and SCCS studies

	**Cohort study**	**SCCS**
	**(N** **=** **105,467)**	**(N** **=** **6775)**
**Exposed (N (%))**	32247 (30.6%)	2839 (41.9%)
**Age (median (range))**	83 (80 – 86)	84 (82 – 87)
**Male gender**	54296 (51.5%)	4189 (61.8%)
**Patients with at least one pneumonia hospitalisation**	6775 (6.4%)	6775 (100%)
**Entered residential aged care facility**	11852 (11.2)	786 (11.6)

The adjusted risk estimates were significant for all post-exposure risk periods (Table 2). The risk of hospitalisation for pneumonia was 3.2 times higher in the first 1 to 7 days after initiation of PPIs compared to unexposed patients. The risk of pneumonia was reduced with more than 30 days continuous treatment but remained significantly higher than unexposed (RR = 1.55 95% CI 1.44,1.67).

**Table 2:** **Exposure to proton pump inhibitors and risk of hospitalisation for pneumonia**; **cohort study adjusted analysis**

**Risk periods**	**Number of hospitalizations for pneumonia**	**Person-years**	**Unadjusted rate ratios**	Adjusted* rate ratios (95% CI)
Baseline unexposed to a proton pump inhibitor				
Unexposed	5598	346016	1.00	1.00
Post-exposure to proton pump inhibitors				
1–7 days	59	1043	3.50 (2.71 – 4.52)	3.24 (2.50 – 4.19)
8–30 days	118	3344	2.18 (1.82 – 2.62)	2.02 (1.68 – 2.42)
>30 days	989	33449	1.83 (1.71 – 1.96)	1.55 (1.44 – 1.67)

*Adjusted for age at entry into the cohort, gender, socioeconomic index of disadvantage for area of residence [11] at study entry, number of co-morbidities (assessed annually using the validated Rx-Risk-V [12] score), season, residential aged-care status, use of tiotropium as a proxy indicator of chronic obstructive pulmonary disease (COPD), use of angiotensin renin system medicines concurrent with frusemide as a proxy indicator of those with heart failure, number of prescriptions, number of prescribers, number of pharmacies, occupational therapy visits and speech pathology services.

### Self-controlled case series

There were 6775 subjects who had at least one hospitalisation for pneumonia during the study period, with 2839 (41.9%) initiated on a proton pump inhibitor. In this study, 61.8% of participants were male and 11.6% entered a residential aged-care facility during the study (Table 1).

There was an increased risk of hospitalisations for pneumonia in the three risk periods following initiation of proton pump inhibitors compared to the baseline period. In both 30 day pre-exposure risk periods there was a statistically significant increased risk of having a hospitalisation for pneumonia compared to the baseline period, however there was no significant risk of hospitalisation 61–120 days prior to a PPI (Table 3).

**Table 3:** **Exposure to proton pump inhibitors and risk of hospitalisation for pneumonia**; **self**- **controlled case**-**series adjusted analysis**

**Risk periods**	**Number of hospitalizations for pneumonia**	**Person-years**	Adjusted* rate ratios (95% CI)	Adjusted** rate ratios (95% CI)
Baseline unexposed to a proton pump inhibitor				
Unexposed	5544	18632	1.0	1.0
Pre-exposure to a proton pump inhibitor				
Pre 1–30 days	476	383	5.24 (4.94 – 5.57)	5.17 (4.86 – 5.49)
Pre 31–60 days	166	353	2.02 (1.85 – 2.20)	2.00 (1.83 – 2.18)
Pre 61–120 days	148	589	1.09 (0.99 – 1.19)	1.07 (0.98 – 1.17)
Post-exposure to proton pump inhibitors				
1–7 days	69	90	3.22 (2.83 – 3.66)	3.07 (2.69 – 3.50)
8–30 days	135	287	2.01 (1.82 – 2.21)	2.00 (1.82 – 2.20)
>30 days	1131	2978	1.67 (1.58 – 1.77)	1.66 (1.56 – 1.76)

* Adjusted for time-varying age and study year only.

**Adjusted for time-varying age, study year, number of co-morbidities (assessed annually using the validated Rx-Risk-V [12] score), season, residential aged-care status, use of tiotropium as a proxy indicator of chronic obstructive pulmonary disease (COPD), use of angiotensin renin system medicines concurrent with frusemide as a proxy indicator of those with heart failure, number of prescriptions, number of prescribers, number of pharmacies, occupational therapy visits and speech pathology services.

## Discussion

This study has shown that using two different study designs, the self-controlled case series and the cohort, that there was an increased risk of hospitalisation for pneumonia after being prescribed a proton pump inhibitor. The self-controlled case series design encourages stratification of the exposure time. This is not usual in cohort studies but was undertaken in this study to compare the rate ratios between studies. The stratification allows for the assessment of the risk of pneumonia, at various *a priori* points in time. This leads to a more detailed understanding of the temporal association between the medicine of interest and the outcome. The rate ratios after continuously taking a proton pump inhibitor for greater than 30 days were similar for both studies (cohort: RR 1.55; 95% CI (1.44, 1.67): SCCS: RR 1.66; 95% CI (1.56, 1.76)). Our findings are higher than those observed in a meta-analysis that identified randomised clinical trials and observational studies that evaluated the association between acid-suppressive drugs and the risk of pneumonia [8]. The meta-analysis of observational studies reported an overall odds ratio of 1.27 (95% CI (1.11, 1.46)) and for the outcome of community acquired pneumonia a odds ratio of 1.34 (95% CI (1.14 -1.57)) [8]. The meta-analysis of the randomised clinical trials found an overall relative risk of 1.22 (95% CI (1.01, 1.48)) [8]. Regardless of the study design utilised, after initiation of proton pump inhibitors the risk of hospitalisation for pneumonia was highest in the first 7 days after initiation. These findings are also consistent with those observed in the meta-analysis of observational studies [8] which found a strong association within the first six days post therapy initiation (OR 3.95; 95% CI (2.86, 5.45)) [8].

As with any statistical method the self-controlled case series method has key assumptions that must be met [2,3]. The first assumption states that recurrent outcome events must be independent, that is, the occurrence of one event must not alter the probability of a subsequent event occurring [2,3]. For some hospitalisations, once a person has experienced that hospitalisation their short-term risk of another occurrence may be increased. Therefore, hospitalisations may cluster within independent episodes. In this study, we included only the first hospitalisation of each episode to ensure the independence of outcome events [2]. The second assumption of the SCCS method is that the occurrence of an outcome event must not alter the probability of subsequent exposures [2,3]. We found a significantly increased risk of a pneumonia hospitalisation prior to proton pump inhibitor exposure in the self-controlled case series design. This may be due to physicians routinely prescribing proton pump inhibitors to patients once they enter hospital. Other studies have identified that a large number of patients initiate these medicines in hospital [13,14]. To account for this we partitioned person-time prior to exposure into separate risk periods. Results show that in the 1–30 days prior to proton pump inhibitor initiation there is over a five times greater risk of hospitalisations for pneumonia than in other non-exposure periods. The risk of hospitalisation is still high 60–31 days before proton pump inhibitors suggesting that initiation after hospital discharge of pneumonia by a GP is still high. However, the risk of hospitalisation had returned to base line after 60 days before PPI initiation. The final assumption is that the occurrence of the event of interest must not censor or affect the observation period [2,3]. While in some cases patients died as a direct result of the pneumonia admission, the majority (87%) of patients in this study were discharged alive from their hospital admission for pneumonia, meaning this assumption is likely to have been met in this study. Farrington et al [15], have shown that this method may be robust to failure of this assumption. Additionally our sensitivity analysis restricted to patients discharged alive from hospital showed that risk estimates in the exposed periods changed only marginally (Table 4). As this may not be the case for all studies, it may be more appropriate to apply one of the two alternative methods that Farrington et al [16,17], have developed.

**Table 4:** **Sensitivity analysis**: **exposure to PPIs and risk of hospitalisation for pneumonia**; **self- controlled case-series adjusted analysis in patients alive at discharge**

**Risk periods**	**Number of hospitalizations for pneumonia**	**Person-years**	Adjusted* rate ratios (95% CI)
Baseline unexposed to a proton pump inhibitor			
Unexposed	4680	16676	1.0
Pre-exposure to a proton pump inhibitor			
Pre 1–30 days	438	347	5.71 (5.37 – 6.07)
Pre 31–60 days	161	319	2.30 (2.11 – 2.51)
Pre 61–120 days	141	530	1.21 (1.11 – 1.33)
Post-exposure to proton pump inhibitors			
1–7 days	52	81	2.87 (2.49 – 3.32)
8–30 days	112	262	1.95 (1.76 – 2.16)
>30 days	944	2786	1.55 (1.46 – 1.65)

* Adjusted for time-varying age and study year only.

The cohort and self-controlled case series study produced comparable relative risk estimates and therefore we are able to draw similar conclusion about the safety of proton pump inhibitors. Bias can be introduced in a cohort study through lack of data on confounders and it can be difficult to deal with the important differences between patients who were and were not prescribed the medicine of interest. The self-controlled case series method, however, may overcome this problem as exposure time is compared to unexposed time in the same patient thereby controlling implicitly for fixed confounders [3,15]. Our sensitivity analyses showed that adjusting for potential time-varying confounders made little difference to the risk estimates in the self-controlled case series design. Two previous studies with similar endpoints demonstrated that the odds ratio did not change after adjustment for these factors [18,19]. The ability of the self-controlled case series method to control for confounding both measured and unmeasured will be of particular practical value in pharmacoepidemiology.

One of the limitations of this study is potential missing data on over the counter proton pump inhibitors medicines and some in-hospital dispensing’s. The pharmacy database utilised in this study contains information on all prescriptions dispensed in private hospitals, public hospital outpatient visits but not for those that were dispensed during a public hospital admission. Data is not collected on over the counter proton pump inhibitors; however the over the counter purchase price is more than double the patient co-payment for prescription medicines and the quantity supplied is half the prescription quantity.

## Conclusions

This study shows that the self-controlled case series method and new-user cohort study design produce similar results. The cohort method was implemented adjusting for multiple confounders and the self-controlled case-series relied solely on its design to adjust for fixed confounders and on the inclusion of the non-exposed group to adjust for time varying factors such as age. These findings suggest that the results of the self-controlled case-series design may be relied upon to investigate the safety of medicines for which limited clinical trial data exist.

### Consent

This study used secondary, de-identified patient data, therefore individual patient consent was not required.

## Competing interests

The research was funded by the Australian Government Department of Veterans’ Affairs (DVA) as part of the delivery of the Veterans’ Medicines Advice and Therapeutics Education Services (Veterans’ MATES) project. The data used in this research is owned by the Department of Veteran’ Affairs and is provided as part of the delivery of the Veterans’ MATES project. DVA reviewed this manuscript prior to submission, but played no role in the design, execution, analysis or interpretation of data, or writing of the paper. The authors have no conflicts of interest to declare.

## Authors’ contributions

ERamsay and ERoughead conceived and designed the study. ERamsay and NP were involved in the analysis of the data. ERamsay, NP, PR and ERoughead were involved in the interpretation of data. ERamsay wrote the initial draft of the manuscript. The manuscript was revised critically for important intellectual content by NP, PR and ERoughead. All authors have given final approval of the manuscript to be published.

## Pre-publication history

The pre-publication history for this paper can be accessed here:

http://www.biomedcentral.com/1471-2288/13/82/prepub

## References

[B1] Suissa S Delaney J The case-crossover study design in pharmacoepidemiology Stat Methods Med Res 2009 18 53 65 10.1177/0962280208092346 18765504

[B2] Whitaker HJ Farrington CP Spiessens B Musonda P Tutorial in Biostatistics: the self-controlled case series method Stat Med 2006 25 10 1768 1797 10.1002/sim.2302 16220518

[B3] Whitaker HJ Hocine MN Farrington CP The methodology of self-controlled case series studies Stat Methods Med Res 2009 18 7 26 10.1177/0962280208092342 18562396

[B4] Glanz J McClure D Xu S Hambidge S Lee M Kolczak M Four different study designs to evaluate vaccine safety were equally validated with contrasting limitations JCE 2006 59 808 818 16828674 10.1016/j.jclinepi.2005.11.012

[B5] Hippisley-Cox J Unintended effects of statins in men and women in England and Wales: population based cohort study using the QResearch database BMJ 2010 340 c2197 10.1136/bmj.c2197 20488911 PMC2874131

[B6] Gulmez S Holm A Frederiksen H Jensen T Pedersen C Hallas J Use of proton pump inhibitors and the risk of community-acquired pneumonia: a population-based case–control study Arch Intern Med 2007 167 9 950 955 10.1001/archinte.167.9.950 17502537

[B7] Laheij R Sturkenboom M Hassing R Dieleman J Stricker B Jansen J Risk of community-acquired pneumonia and use of gastric acid-suppressive drugs JAMA 2004 292 16 1955 1960 10.1001/jama.292.16.1955 15507580

[B8] Eom C-S Jeon C Lim J-W Cho E-G Park SM Lee K-S Use of acid-suppressive drugs and risk of pneumonia: systematic review and meta-analysis CMAJ 2011 183 3 310 319 21173070 10.1503/cmaj.092129 PMC3042441

[B9] Gadzhanova SV Roughead EE Mackson JM Initiation and duration of proton pump inhibitors in the Australian veteran population Intern Med J 2012 42 E68 73 10.1111/j.1445-5994.2010.02259.x 20492006

[B10] Suissa S Immortal time bias in pharmacoepidemiology Am J Epidemiol 2008 167 4 492 9 10.1093/aje/kwm324 18056625

[B11] Australian Bureau of Statistics (ABS) Information paper: census of population and housing Socio-economic indexes for areas, Australia, 2001 ABS cat. no. 2039.0 2003 Canberra: ABS

[B12] Sloan KL Sales AE Liu CF Fishman P Nichol P Suzuki NT Construction and characteristics of the RxRisk-V: a VA-adapted pharmacy-based case-mix instrument Med Care 2003 41 6 761 74 PubMed PMID: 12773842 12773842 10.1097/01.MLR.0000064641.84967.B7

[B13] Thomas L Culley E Gladowski P Goff V Fong J Marche S Longitudinal analysis of the costs associated with inpatient initiation and subsequent outpatient continuation of proton pump inhibitor therapy for stress ulcer prophylaxis in a large managed care organisation J Manag Care Pharm 2010 16 2 122 29 20178397 10.18553/jmcp.2010.16.2.122 PMC10437701

[B14] Zink DA Pohlman M Barnes M Cannon ME Long-term use of acid suppression started inappropriately during hospitalization Aliment Pharmacol Ther 2005 21 1203 9 10.1111/j.1365-2036.2005.02454.x 15882240

[B15] Farrington C Whitaker H Semiparametric analysis of case series data Appl Statist 2006 55(Part 5) 553 94

[B16] Farrington C Anaya K Whitaker HJ Hocine M Douglas I Smeeth L Self-controlled case series analysis with event-dependent observation periods American Journal of Statistical Association 2011 106 494 417 26 10.1198/jasa.2011.ap10108

[B17] Farrington CP Whitaker HJ Hocine MN Case series analysis for censored, perturbed or curtailed post-event exposures Biostatistics 2009 10 1 3 16 18499654 10.1093/biostatistics/kxn013

[B18] Laheij RJ Van Ijzendoorn MC Janssen MJ Jansen JB Gastric acid-suppressive therapy and community-acquired respiratory infections Aliment Pharmacol Ther 2003 18 8 847 51 PubMed PMID: 14535879 10.1046/j.1365-2036.2003.01744.x 14535879

[B19] Gulmez SE Holm A Frederiksen H Jensen TG Pedersen C Hallas J Use of proton pump inhibitors and the risk of community-acquired pneumonia: a population-based case–control study Arch Intern Med 2007 167 9 950 5 PubMed PMID: 17502537 10.1001/archinte.167.9.950 17502537

